# Nerve regeneration by interferon intervention in aging brain

**DOI:** 10.15252/emmm.202217307

**Published:** 2023-03-10

**Authors:** Fanwen Wang, Duanqing Pei

**Affiliations:** ^1^ Laboratory of Cell Fate Control, School of Life Sciences Westlake University Hangzhou China; ^2^ College of Life Sciences Zhejiang University Hangzhou China

**Keywords:** Immunology, Neuroscience, Stem Cells & Regenerative Medicine

## Abstract

Neural stem cells (NSCs) are shielded from viral infection by interferon (IFN) defense. As individuals age, activation of NSC decreases with a significant decline of stemness marker Sex‐determining region Y box 2 (Sox2) while IFN signaling enhances (Kalamakis *et al*, 2019). Given that low‐level type‐I IFN under normal physiological conditions can promote dormant hematopoietic stem cell differentiation (Baldridge *et al*, 2010), whether there is an inner connection between IFN signaling and NSC function remains elusive. In this issue of *EMBO Molecular Medicine*, Carvajal Ibanez *et al* (2023) reveal that IFN‐β, a type‐I interferon, induces cell‐type‐specific interferon‐stimulated genes (ISGs) and regulates global protein synthesis by orchestrating mTOR1 activity and stem cell cycle that retain NSCs at the G_0_ phase and repress Sox2 expression. As a consequence, NSCs exit the activation state and become inclined to differentiation.

## Graphical abstract

F. Wang and D. Pei discuss the study by Carvajal Ibanez *et al* (in this issue of *EMBO Mol Med*) that investigates the connection between IFN signaling and neural stem cell functions.

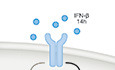

Neural stem cells, located in the ventricular–subventricular zone (vSVZ) and subgranular zone (SGZ), give birth to olfactory bulb interneurons and granule cells throughout adult life to maintain the odor discrimination and hippocampus homeostasis, respectively. The NSC‐niche‐surrounding rich blood vessels increase the risk of exposing NSCs to various pathogens. Neural stem cells could benefit from continuously expressed intrinsic ISGs to be protected from virus invasion (Wu *et al*, 2018). Interferon can be classified into three different types based on antigenicity and biological properties. Type‐I, including IFN‐α and IFN‐β, is mainly produced by the epithelial cells and fibroblasts, respectively. Type II includes IFN‐γ produced by natural killer cells and activated T cells. Type III is IFN‐λ. Circulating IFNs are limited to the immune system due to the presence of the blood–brain barrier, while a small amount of exogenous IFNs can still penetrate the brain through areas where the blood–brain barrier is more permeable. Type I and type II IFN activate downstream JAK/STAT signaling through binding to IFNAR and IFNGR, which ubiquitously exist in neurons and glial cells, respectively. IFN‐β regulates the removal of myelin residues and dead cells in the central nervous system, thereby reducing inflammation. Type III IFN receptors locate on immune cells and epithelial cells and defend against viruses mainly through immune cell recruitment. Once infected or injured, NSCs become activated and differentiate into neurons and glial cells (Delgado *et al*, 2021). As aging proceeds, however, the activation and progenitor productivity of NSCs decrease while the IFN signaling pathway increases. Type‐I IFN may also reduce cognitive behavioral ability during aging and in virus‐infected individuals. But the complete absence of IFN‐β would cause neurodegenerative diseases. Previous studies focus on transcription control of ISGs while the underlying molecular mechanism of IFN regulation on stemness factors is mysterious. Understanding how the internal ISGs and the exogenous IFN response coordinate is an urgent need for regenerative therapy during aging.

In this study, NSCs isolated from mouse brains are subjected to single‐cell RNA sequence and Ribo‐seq. These isolated mouse NSCs show transcriptomic heterogeneity and can be classified into five clusters: The dormant NSCs state 1 (Aqp4^+^ S100b^+^ Egfr^+^), dormant NSCs state 2 (Aqp4^+^ Egfr^+^), activated NSC (Egfr^+^), transient amplifying progenitors (DCX^+^ Egfr^+^ Ki67^+^), and the final neuroblast (DCX^+^; Urban *et al*, 2019) with different IFN response intensity. Neural stem cells and their terminal‐differentiated neurons from both young and old mouse brain exhibit ubiquitous‐IFN‐I receptor‐dependent IFN response, which is absent in neural progenitor cells. At the same time, intrinsic ISGs still exist in IFNAGR knockout mouse NSCs, suggesting stemness confers an intrinsic interferon response. Previous studies show that embryonic stem cells exert antiviral activity mainly through intrinsic ISGs. Neural stem cells do not produce IFN‐I when exposed to viruses or treated with poly(I:C), and respond only weakly to exogenous IFN (Hong & Carmichael, 2013). This study shows, however, NSCs not only to express intrinsic ISGs but also to present strong exogenous IFN response that accompanies the entire mouse life span. On the other aspects, NSCs treated with IFN‐β are arrested under the G_0_ phase and exhibit an early‐upregulated followed by late‐downregulated global mRNA translation. There is a mild increase in global protein synthesis after treatment with IFN‐β for 2 h and a profound decrease after 14 h. By combining polysome profiling and Western blot, they find that the early IFN‐β response is mainly modulated by activity‐promoted mTOR, as a consequence of PI3K/Akt signaling inhibition on T1462 phosphorylation of TSC2, while the late IFN‐β response is mainly modulated by S51 phosphorylation of eiF2α and activity‐repressed mTOR due to free TSC2. In a nutshell, IFN‐β can arrest NSCs at the G_0_ phase and biphasically controls NSC transcriptome through mTOR and eiF2α.

In addition, this biphasic regulation can also affect the activation status of NSCs. Carrasco‐Garcia *et al*, (2019) found that the declined Sox2 expression in the aging brain can induce NSCs into dormancy. This suppression of Sox2 expression can also be observed in NSCs treated with IFN‐β. Here, Carvajal Ibanez *et al* verified that transient mTOR increase induced by IFN‐β regulates the pyrimidine‐rich motif present in the 5'UTR of Sox2 and causes NSC stemness loss. But how do IFNs fine‐tune the adult vSVZ's neurogenesis across an individual's life span? The authors examine the activation rate and self‐renewal ability of NSCs (Prom1^+^ GLAST^+^) isolated from IFNAGR^KO^ or IFNAGR^WT^ mice at the age of 2, 6 ~ 7, and 22 months. Mathematical modeling further predicts that IFN can influence NSC stemness and progenitor cell production, consistent with the effect of IFN promotion on hematopoietic stem cell differentiation (Baldridge *et al*, 2010). Model simulation results suggest IFN intervention may be good for neurogenesis for specific advanced ages (older than 350 days) but not beneficial for all stages. Collectively, the fine‐tuned network dominated by interferon repression on Sox2 level, as well as the induction of NSC quiescence that is orchestrated by uncoupling cell cycle and mTOR activity are indispensable for neural stem cell physiological function throughout mouse life (Fig 1).

**Figure 1:**
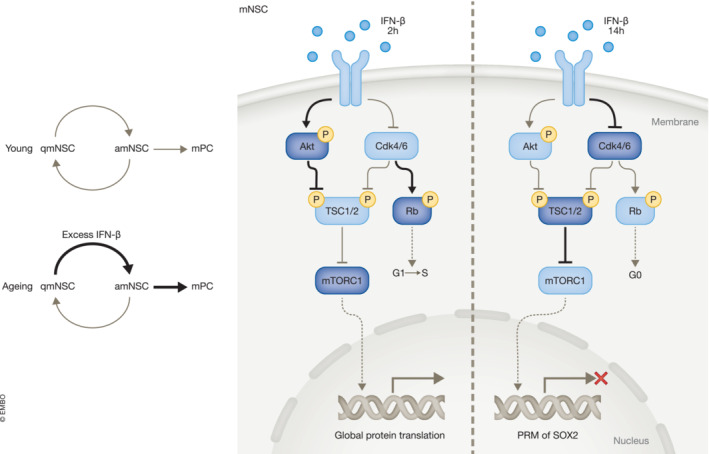
Interferon‐beta represses SOX2 level to induce mouse neural stem cell exit activation state by fine‐tuning mTORC1 activity and cell cycle In the young mouse brain, the activation rate and self‐renewal of NSCs are in dynamic equilibrium to maintain homeostasis. The increased IFN‐IFN‐β levels, along with aging, can biphasically induce a transient up‐ and late‐ downregulation of global protein synthesis through modulation of mTORC1 activity and a concomitant gradual inhibition of the cell cycle. MTORC1 inhibits Sox2 expression and induces quiescence of neural stem cells. These findings suggest that IFN inference would be beneficial for the aging brain. amNSC, activated mouse neural stem cell; IFN, interferon; mPC, mouse progenitor cell; qmNSC, quiescent mouse neural stem cell.

Neural regeneration has always been the focus of neurodegenerative and aging‐related brain diseases. Aging‐related metabolism changes, inflammation infiltration, and accumulated neural genome mutations since birth, etc., will directly or indirectly break the homeostasis of nervous system. Fortunately, scientists now have found ways to generate neurons by transdifferentiating glia or activating quiescent NSCs. The study by Carvajal Ibanez *et al* unveils the molecular mechanism of the fine‐tuned regulation by IFN that promotes NSC to exit activation state and to enter into differentiation state without affecting antiviral response, which, in fact, is slightly strengthened when the IFN receptor is blocked. These findings identify IFN as a potential therapeutic target for neuron production in the aged brain. Given that IFN response is suppressed in tumor progression, treatment with IFN for tumor stem cells worths a shot. It is worth noting that the transcriptome differences between human NSCs and mouse NSCs may lead to a different mechanism. In addition, since data produced by this study come from an *ex vivo* mouse NSC culture system, it is even better to analyze data from *in vivo* mouse and human postmortem samples. Intriguingly, IFN‐β was reported to be an aging‐related driver that promotes aging by activating the P53 pathway in DNA‐damaged cells (Yu *et al*, 2015). How NSCs escape aging under IFN stimulation is also a problem that needs to be resolved.
